# Enhancing three-phase induction motor reliability with health index and artificial intelligence-driven predictive maintenance

**DOI:** 10.1098/rsos.241946

**Published:** 2025-05-28

**Authors:** Felipe Lima Aires, Gabriel Dias Galeno, Fernando Nunes Belchior, Antonio Melo Oliveira, Julian David Hunt

**Affiliations:** ^1^ Faculty of Science and Technology, Federal University of Goias, Aparecida de Goiania, Goias, Brazil; ^2^ School of Electrical, Mechanical and Computer Engineering, Federal University of Goias, Goiania, Goias, Brazil; ^3^ Environmental Sciences and Engineering, King Abdullah University of Science and Technology, Thuwal, Saudi Arabia

**Keywords:** predictive maintenance, artificial intelligence, induction motor, time-series forecasting, power quality

## Abstract

The aim of this work is to assist in the maintenance of three-phase induction motors by creating a health index for this equipment. The proposed approach is based on power quality concepts, the creation of an algebraic algorithm to determine the health index and the use of artificial intelligence algorithms for modelling time series, such as Autoregressive Integrated Moving Average and Facebook Prophet, to predict the future health of the motor based on its historical data. The use of historical data makes it possible to anticipate potential failures and guide predictive maintenance strategies, helping to reduce costs and minimize unplanned downtime. The study examines various causes of failure in three-phase induction motors, analysing some of the most recurrent failures, their implications and the resulting impacts on the performance of the three-phase induction motor.

## 1. Introduction

In today’s industrial systems, operational efficiency and predictive maintenance have become crucial aspects to ensure the reliability and durability of equipment. In the industry, three-phase induction motors play a fundamental role in all branches of the sector [1,2]. These motors are ubiquitous in industrial applications due to their robustness, reliability and versatility. They are commonly used in various processes such as conveyor systems, pumps, compressors, fans and machine tools, providing the necessary mechanical power for a wide range of manufacturing operations. In Brazil, approximately 20 million three-phase induction motors are in operation, consuming around 144 GWh per year, which accounts for 25% of the country’s total energy consumption [3]. The energy wasted by these motors, generated at a cost ranging from 66 to 254 USD per MWh [4], depending on the energy source, leads to significant financial burdens for consumers and an even greater expense for industries. These industries not only bear the cost of wasted energy but also face unplanned shutdowns caused by motors operating under poor conditions.

With the ongoing push towards digitalization, industrial sectors are increasingly adopting solutions inspired by Industry 4.0 principles, focusing on diagnostic and predictive tools that leverage data to improve both design and service efficiency [5,6]. By integrating these technologies, companies can better anticipate and address maintenance needs, avoiding the risks associated with unplanned interventions. When maintenance is not properly scheduled, it can result in unexpected costs due to unsynchronized production and maintenance activities. This can lead to unscheduled downtimes, increased operational expenses and loss of productivity due to inefficient use of labour, equipment and facilities caused by equipment malfunction [7,8]. These disruptions significantly contribute to the total life cycle costs of industrial products, where maintenance expenses are a major factor [9]. By improving maintenance management, especially through predictive methods, companies can not only minimize disruptions but also lower overall life cycle costs by ensuring more consistent and efficient production processes. The importance of equipment maintenance in a production system and the knowledge of motor operating conditions are well-known and widely explored in the literature. Several studies focus solely on the importance of maintenance and its impact on operational efficiency, emphasizing traditional methods for understanding equipment conditions [10–15]. Additionally, other research investigates the use of artificial intelligence (AI) and machine learning in these analyses [16,17], highlighting the potential of these advanced technologies to further optimize asset management and enhance efficiency.

This paper presents an approach for defining a health parameter for three-phase induction motors, based on concepts of power quality. The proposed method is implemented with easily acquired electrical data, and the algorithm, being simple and based on basic mathematical concepts, can be applied using few computational resources. Additionally, the integration of algorithms for time-series modelling is proposed, specifically the Autoregressive Integrated Moving Average (ARIMA) algorithm, and the Facebook Prophet algorithm for future prediction of motor health based on its historical data [18–20]. All data were generated through simulations using MATLAB/Simulink software [21,22], where concepts of power quality and international standards [23–25] were used to identify the operating limits of the simulated motors. Additionally, data preprocessing was conducted to prepare them for manipulation by the operational time-series algorithms. The use of historical data allows for anticipating potential failures and guiding predictive maintenance strategies, contributing to cost reduction and minimizing unplanned downtime. Through this approach, it is expected not only to enhance the reliability of three-phase induction motors but also to establish a paradigm for the effective implementation of health monitoring and prediction systems in industrial environments as the transformation of raw data into actionable information involves three main stages: data collection, data to information transformation and information management and integration [5]. The method proposed in this paper covers the first two stages.

## 2. Theoretical background

Early and accurate detection of faults in electric motors is of utmost importance for preventive maintenance and ensuring the reliable operation of electrical equipment in industrial systems. The lack of timely fault detection can result in substantial repair costs, unplanned downtime and safety risks. Therefore, the development of AI systems for automatic fault detection in motors has been a significant area of research in recent decades, where the main goal of the algorithms being investigated is to train a system to identify behavioural patterns in motors that may lead to a fault, thereby providing an opportunity for predictive maintenance planning.

### 2.1. Basic concepts about three-phase induction motors

#### 2.1.1. Three-phase induction motors

To identify faults in a motor, it is necessary to first understand its operation. The first step is to be acquainted with the various types of electric motors available. This foundational knowledge is crucial in motor analysis, allowing for a deeper understanding of how to diagnose faults and optimize performance. In document [26], WEG provides a diagram depicting many types of electric motors. Specifically, the three-phase induction alternating current (AC) motor with a squirrel-cage rotor is identified for analysis in this work. This motor type is recognized for its broad application in both industries and commercial installations, thanks to its simplicity, reliability and efficiency, making it one of the most widely used in industries and commercial installations. As observed in [26], its structure is composed of the following two main parts: the stator and the rotor. Understanding the composition of these parts is essential to properly diagnosing potential faults in the motor.

When the motor is connected to the three-phase electrical network, the stator coils are energized by the alternating current. This current generates a rotating magnetic field that, in turn, induces an electric current in the rotor. This current in the rotor generates another magnetic field. The interaction between these two fields creates electromagnetic forces that drive the rotation of the rotor, causing the motor to operate [27]. The rotor of the squirrel-cage motor is composed of aluminium or copper bars arranged axially and interconnected by rings at the ends, forming a cage. This simple and robust structure ensures low production costs and high reliability for the motor. The motor model being used in the study is an asynchronous motor, where the rotor’s speed lags slightly behind the rotating magnetic field generated by the stator currents. With this principle, it is possible to understand some concepts related to the induction motor, namely synchronous speed, slip and torque. The synchronous speed of the motor is defined by the rotation speed of the rotating magnetic field, and this speed depends on the number of poles of the motor and the line frequency (*f*). The frequency represents the number of cycles per second that the electric field undergoes. The unit used to represent synchronous speed is revolutions per minute (RPM), and it can be calculated according to equation (2.1), where $W_{s}$ is the synchronous speed (in RPM), f is the frequency of the line (in Hz) and p is the number of poles of the motor.


(2.1) $$W_{s}=\frac{\left(120.f\right)}{p}.$$

Unlike synchronous motors where the rotor and magnetic field rotate at the same speed, in asynchronous motors, the rotor’s speed lags behind the rotating magnetic field. This difference in speed is called slip(s). As the rotor conductors move through the magnetic field, currents are induced in the rotor winding according to the laws of electromagnetism. This interaction between the induced currents and the magnetic field generates the motor torque. The slip percentage ‘*s*’ is a dimensionless quantity and can be calculated according to equation (2.2), where $W_{n}$ is the rotor speed (in RPM).


(2.2) $$s\left(\%\right)=100.\frac{\left(W_{s}-W_{n}\right)}{W_{s}}.$$

Torque is a physical quantity that describes the tendency of a force applied to an object to rotate it or cause it to rotate around a point or axis. In an induction motor, the motor generates torque to overcome the load and keep it rotating. The greater the load, the greater the torque required to move it. To achieve higher torque, the difference between the field and rotor speeds must be greater, so that the induced current and the generated field are more intense. Therefore, as the load increases, the motor speed decreases. When the load is zero (motor unloaded), the rotor rotates practically at its synchronous speed. Torque is a quantity represented in Newton metre (Nm), and it can be calculated according to equation (2.3), where τ is the motor nominal torque (in Nm) and P is the motor electrical power (in Watts).


(2.3) $$\tau \;=\frac{P}{2\pi .\frac{W_{n}}{60}}.$$

With a thorough understanding of the principles and concepts related to electric motors, it becomes possible to explore the associated failures. This work focuses on the analysis of carefully selected faults, considered particularly relevant within the scope of this project. Next, some of the addressed failures will be detailed.

#### 2.1.2. The failures related to the three-phase induction motor

As three-phase induction motors are widely used in the industry, and due to this extensive usage, there is a considerable amount of literature addressing the faults associated with these devices. Several methods have been developed for detecting faults in three-phase induction motors, aiming at the implementation of predictive maintenance systems [28–34]. This aims to reduce financial losses resulting from equipment replacement or motor downtime. Despite the extensively explored topic, the algorithms and methods traditionally used to detect faults were restricted to only one type of fault, such as Motor Current Signature Analysis (MCSA) methods, Extended Park Vector Approach or partial discharge [2,35]. A study made by [2] shows which methods were used for which fault detections. Within the outlined faults, this paper will explore issues related to power quality and mechanical aspects, such as voltage harmonics, voltage imbalance, long-duration voltage variations, short circuits and coupling failure.

In an ideal electrical system, the supply voltages should be, according to the supply contract, perfectly sinusoidal and balanced. However, in practice, voltage and current signals often exhibit waveform distortions, and by analysing these distorted waveforms, Fourier methods can decompose them into perfectly sinusoidal waves that are multiples of the fundamental frequency. These waves, known as harmonics, occur when the frequency is different from the fundamental frequency [36–38]. This phenomenon is typically caused by the operation of loads with nonlinear characteristics. Nonlinear devices typically produce odd harmonic distortions, with those closer to the fundamental frequency having a higher magnitude. This behaviour is common in most electrical systems. Even harmonics, on the other hand, are rare and typically arise from malfunctioning nonlinear loads, producing half-wave asymmetries [39,40]. In the context of three-phase induction motors, the analysis of even harmonics is not justified. These motors, being part of balanced systems with linear characteristics, rarely exhibit even harmonics, which are more common in single-phase or unbalanced systems. Additionally, the analysis of odd harmonics that are multiples of 3 is unnecessary since the line voltage in three-phase systems, composed of sinusoidal voltages phased 120 degrees apart, tends to cancel out third-order harmonics due to the phase shift [41,42]. In the calculation of odd harmonics not multiples of 3, which are fundamental to this research, Prodist [25] serves as the reference. This standard governs the acceptable parameters for electrical energy quality in Brazil, and the following equations will serve as the basis for our harmonic analysis. To find the THDv (total harmonic distortion of voltage for odd non-multiple-of-3 components), equation (2.4), as displayed in [25], is used, where *h* are the odd harmonic orders, not multiples of 3 and *hi* are the maximum odd harmonic order, not multiple of 3.


(2.4) $$\text{THDv}\;\%=\frac{\sqrt{\sum_{h=5}^{hi}Vh²}}{V1}\;x\;100.$$

According to module 8 of Prodist [25], voltage imbalance occurs when there is a difference in amplitudes between the three-phase voltages in a specific three-phase system or when there is an electrical phase shift different from 120° between the phase voltages of the same system. This imbalance can lead to issues in electrical equipment, affecting the efficiency and stability of the electrical network. It can cause excessive currents in motors, resulting in losses, increased temperature and reduced lifespan. The origins of this imbalance are usually related to unevenly distributed single-phase loads in distribution systems, generating negative sequence voltages in the circuit. Another common cause of the problem is fuse blowouts in one phase of a bank of three-phase capacitors [1,43]. The issue worsens when consumers powered by three-phase electrical systems have uneven load distribution in their internal circuits, causing unbalanced currents in the utility’s network. For the calculation of the voltage imbalance, equation (2.5) [25,38] is used, where ‘*V*−’ is the negative sequence phase-to-phase voltage, and ‘*V*+’ is the positive sequence phase-to-phase voltage.


(2.5) $$\text{FD}\%\;=\frac{V_{-}}{V_{+}}.100.$$

When addressing voltage variation issues, long-duration effects lasting more than 3 min are significant, as they fall under the category of steady-state disturbances [25,38]. These disturbances are characterized by deviations in the root-mean-square (RMS) value of voltage and the frequency of the electrical system. Such variations may be related to overvoltage or undervoltage situations, as well as prolonged faults. Typically, these conditions are not caused by inherent system problems but result from fluctuations in energy demand or switching manoeuvres. These variations are typically represented and studied through graphs showing the variation of the RMS voltage signal over time [36,38]. For instance, in the standard [23], parameters for undervoltage and overvoltage are defined. Monitoring these voltage variations is crucial for ensuring the stability and proper functioning of electrical systems, as adherence to established standards helps mitigate potential risks associated with prolonged deviations from nominal operating conditions.

The most frequently observed occurrence in electrical systems is the short circuit, which manifests through the flow of intense currents through all energized components. This event causes severe voltage disturbances throughout the electrical network, often resulting in irreversible damage to both the system itself and consumer installations [43,44]. A short circuit can arise in various forms, particularly in motor stators where the conductors of each phase present in the motor stator are confined in a small space. A small fault in the insulation of these conductors can lead to a short circuit between adjacent conductors [1,2,42]. Understanding the causes and potential forms of short circuits is crucial for implementing effective preventive measures, thereby reducing the likelihood of severe damage to electrical systems and associated installations.

Misalignment is a common occurrence when it comes to the connection between a load and the motor shaft or transmission equipment, often leading to unwanted vibration. Both parallel and angular misalignment of the shafts can cause radial and axial vibrations, which are difficult to avoid due to factors like improper assembly and potential movement of the base or foundation [1,45–47]. This misalignment significantly affects the rotor system’s dynamics, leading to undesired responses such as coupling deflection and premature bearing wear. The effects of misalignment are evident through increased motor temperature, excessive vibration and altered torque, which have been thoroughly documented in various studies [40,46–50]. Therefore, it is of utmost importance to identify and correct any misalignment as quickly as possible to ensure the safe and efficient operation of the machine or equipment in question, as addressing these issues can prevent further damage and extend the equipment’s lifespan.

#### 2.1.3. Effects of failures on a three-phase induction motor

Three-phase induction motors are designed to operate reliably, maintaining consistent performance over time. During the production process, they undergo testing in an environment that simulates their nominal conditions. However, it is important to note that this test environment is not always reproducible in the location where the motor will be used. Due to this environmental variation, motors may exhibit parameters different from those tested during manufacturing. This discrepancy can result in operational conditions that generate acceptable parameters, but it can also lead to situations where the parameters become unacceptable. In these cases, mechanical, electrical or thermal stresses may occur in the motor, resulting in wear of important components such as insulation and bearings. This mechanical wear, in turn, is a precursor to failures that can compromise the motor’s operation. Some well-known consequences of failures in three-phase motors include increased temperature, fluctuations in developed electromechanical torque and motor vibrations [46,47,49,51]. However, collecting data on these quantities during motor operation is not always an easy or economically viable task [2,38]. For this reason, collecting current and voltage parameters can provide an advantage when analysing motor health.

The operating temperature of a motor is significantly influenced by motor failures, making it a critical parameter for monitoring. Failures in an induction motor typically lead to increased losses, which manifest as thermal radiation, thereby raising the motor’s temperature. Research by [52,53] provides detailed insights into how different motor failures, such as misalignment, can cause stresses and unnecessary friction on the rotor, directly impacting temperature. Those impacts occur because when the shafts are not perfectly aligned, there is an uneven distribution of loads and forces within the system. As a result, the motor is subjected to additional stresses and unnecessary friction, leading to an increase in heat production. Furthermore, [54] shows that even a minor voltage imbalance can result in a significant temperature rise in three-phase induction motors. These findings underscore the importance of monitoring motor temperature as a reliable indicator of potential failures, emphasizing that both mechanical issues like misalignment and electrical issues like voltage imbalance can lead to harmful increases in temperature, thus compromising motor efficiency and longevity.

Another well-documented consequence of motor failures is vibration, often linked to misalignment in the motor’s centre of mass and anomalies in the motor’s electromagnetic torque [46,49,51,52,54–57]. While electromagnetic torque anomalies are typically caused by issues in power quality, the shift in the motor’s centre of mass is caused by improper coupling of the load or improper motor base installation. Studies by [45,58] identify shaft misalignment as a primary cause of unwanted vibrations in industrial machinery, as it generates reaction forces in the coupling that significantly affect the equipment. A notable phenomenon is the restoring moment present in the coupling, which tends to reposition the motor shaft. However, in cases of misalignment, this restoring moment contributes to the vibration of the device. The magnitude of these forces and moments, driven by the degree of misalignment, directly influences motor torque behaviour, as variations in misalignment intensity affect torque response [42,58].

When the phase voltages applied to induction motors vary in both magnitude and phase angle, the primary consequence is the distortion of the rotating magnetic field, leading to an operation similar to that of a motor with a non-uniform air gap. Consequently, increased misalignment results in a higher torque requirement to maintain equipment operation. This increase in torque consumption not only reduces the operational efficiency of the machine but can also lead to premature wear of components and ultimately to a decrease in equipment lifespan [1,59]. Furthermore, the direct effects on the energy supplied to the motor cause oscillations in torque due to their impact on the electromagnetic fields produced by the windings. More specifically, these faults cause an imbalance in the magnetic flux of the electric motor, resulting from asymmetries in the magnetic field distribution relative to the windings. This imbalance leads to non-uniform magnetic forces affecting the motor’s moving parts.

**Figure 1.**
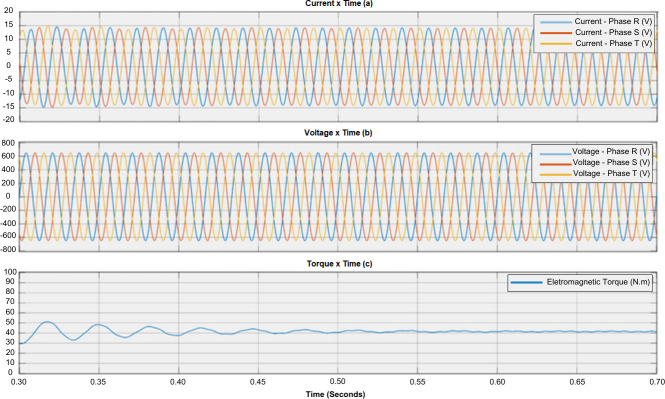
Motor performance under nominal conditions. (a) Current waveform, (b) voltage waveform and (c) torque waveform.

**Figure 2.**
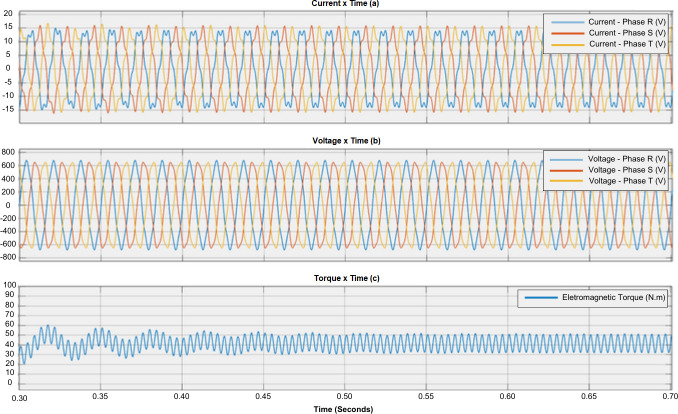
Effect of fifth harmonic voltage on motor performance. (a) Current waveform, (b) voltage waveform and (c) torque waveform.

**Figure 3.**
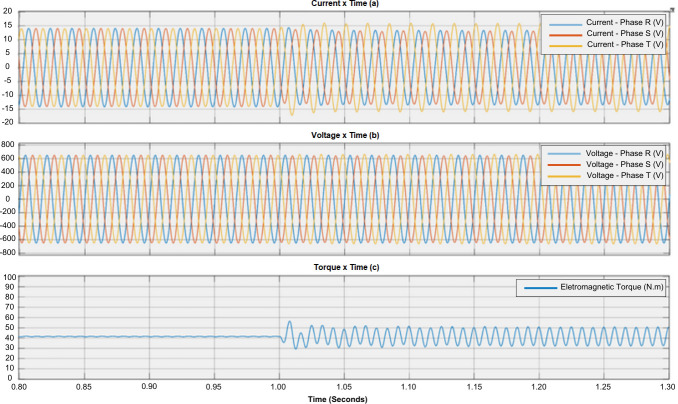
Effect of voltage imbalance on motor performance. (a) Current waveform, (b) voltage waveform and (c) torque waveform.

The normal operation of the induction motor can be compared with a motor experiencing failure through the sequence. Figure 1 shows the relationship between phase currents and torque during normal operation. Figures 2 and 3 illustrate the impact of failures on electromagnetic torque. It can be observed that different failures have varying levels of impact on the torque [21].

Another widely used parameter for fault detection is the phase currents in an MCSA [2,43,48]. This is because the current in the phases of a three-phase induction motor is sensitive to various factors, including the presence of short circuits and voltage imbalance exceeding 2% [25]. When a short circuit occurs between the phases, the system impedance decreases, resulting in a sudden increase in current. This increase can cause imbalances, leading to variations in current intensities in each phase [60]. Moreover, during waveform distortions in voltage, the motor’s natural response is to adjust its current to accommodate these variations. For example, if there are voltage fluctuations, the motor may adjust its current to maintain constant power. These current adjustments can result in variations in current intensity in the motor phases [56].

### 2.2. Matlab simulation using Simulink

The Matlab software is a mathematical modelling tool that can be used in various contexts. Matlab provides versatile function creation and the application of mathematical tools, especially geared towards simulations. Within this platform, Simulink stands out, being a modular tool that offers a variety of ready-to-use models [21,22]. This work focuses on the application of simulated models of three-phase induction motors and sources in Simulink. These models were employed to simulate the performance of these motors under various faulty operating conditions. This approach aims to eliminate the initial need to subject real motors to stressful situations, minimizing physical damage and optimizing resources. The use of simulated models not only allows for an in-depth analysis of motor behaviour but also eliminates the need for the use of real equipment in this initial study.

### 2.3. Artificial intelligence and machine learning

AI refers to the theory and development of computational systems that can perform tasks that typically require human intelligence, including visual information interpretation, speech recognition, decision-making and translation between different languages [61,62]. In the field of AI, there are three main groups of computational models: supervised models, unsupervised models and reinforcement learning. Each model has its own application, and, therefore, this should be considered when choosing the algorithm.

In supervised learning models, the system receives explicit feedback for its predictions, which can be divided into classification and regression methods. Some of the widely recognized algorithms in this category include K-Nearest Neighbours, Decision Tree, Support Vector Machine, Logistic Regression, Artificial Neural Networks and Naive Bayes, among others [61]. On the other hand, unsupervised learning algorithms operate without the need for feedback for their predictions, focusing on discovering latent patterns in the data. Some notable examples of unsupervised learning algorithms include K-Means, the Self-Organizing Model and Latent Dirichlet Allocation, among others. Additionally, the K-Means clustering algorithm is frequently used in preprocessing and anomaly detection steps [61]. In the literature, these algorithms and methodologies are also widely applied for fault diagnosis and prediction, as they excel in handling large datasets, enabling real-time anomaly detection and adapting to various sectors such as manufacturing, energy and healthcare. This versatility is expected given AI’s strengths in detecting and extracting complex patterns from data, even in highly dynamic environments [63–67].

Given the context of this project, the adoption of a supervised algorithm is more coherent, since the proposal is to create a database with parameters carefully defined as targets to be achieved by the algorithm. In this perspective, there is a need to carefully select the algorithms that best suit the obtained data. After evaluating the available options, ARIMA and Facebook Prophet were chosen due to their accessibility, ease of use and widespread adoption in the field of time-series forecasting [20,68].

Both ARIMA and Facebook Prophet are widely recognized for their simplicity in parameterization and configuration, making them particularly suitable for users with varying levels of expertise. These algorithms are favoured for their user-friendly implementation and the vast amount of learning resources available online, which significantly reduce the complexity of applying them to practical scenarios. Additionally, their ability to effectively capture trends, seasonality and other time-series patterns aligns well with the goals of predictive maintenance analysis, where the aim is to forecast future data based on historical patterns.

Their widespread use, coupled with the availability of tutorials, documentation and community support, makes ARIMA and Facebook Prophet the ideal choices for this project, ensuring not only the feasibility of implementation but also the reliability of results.

#### 2.5.1. ARIMA machine learning model

ARIMA, which stands for ‘Autoregressive Integrated Moving Average’, is a widely used statistical model for time-series analysis and forecasting. It combines autoregressive (AR), moving average (MA) and differencing (I) components to model temporal patterns in data [20,69]. ARIMA is extensively applied in various fields, including financial forecasting, demand forecasting, climatological analysis and system performance monitoring, among others. This model is particularly effective when the time series exhibits temporal patterns and when short-term forecasts are required. It is important to note that the algorithm requires the data to be stationary, meaning its statistical properties, such as mean and variance, must remain constant over time. This is because the model assumes that the relationships between observations are constant over time. If the time series is not stationary, meaning its statistical properties are changing, the ARIMA model may not be able to adequately capture these changes [20].

#### 2.5.2. Facebook Prophet machine learning model

Facebook Prophet is a time-series forecasting model developed by Facebook, designed to simplify and automate the forecasting process in various contexts. Released in 2017, Prophet is known for its ease of use, flexibility and effectiveness in handling seasonal patterns [19,70]. The Facebook Prophet algorithm finds applications across a wide range of sectors and contexts. For instance, retail and e-commerce companies use Prophet to anticipate product demand and adjust inventory levels, and financial companies use the algorithm to forecast market trends, analyse the behaviour of financial assets and conduct risk assessments. Unlike ARIMA, Prophet can be used for medium- and long-term forecasts, making it a more accurate and versatile tool.

### 2.6. State of art

The occurrence of failures in electric motors is widely recognized in the literature as a critical issue due to its significant impact on the reliability and efficiency of industrial systems. The study presented in [71] offers a comprehensive review of traditional fault detection techniques, emphasizing conventional monitoring and diagnostic methodologies.

With advancements in AI, numerous studies have investigated the integration of AI techniques into motor monitoring systems to improve fault identification and mitigation. However, most research in this field remains focused on fault detection, which primarily involves identifying issues after they have already manifested [72–83]. This reactive approach, typically based on classification algorithms, enhances corrective maintenance by reducing repair costs and frequency through faster and more efficient fault identification. Intelligent monitoring enables prompt responses, facilitating informed decision-making to mitigate operational disruptions.

On the other hand, the application of AI-driven predictive maintenance, which leverages time-series forecasting algorithms, remains relatively underexplored in the literature. Existing approaches often present limitations that hinder their practical applicability. In some cases, fault prediction is performed with a very short forecast horizon, restricting the feasibility of implementing effective maintenance strategies, as demonstrated in [84,85]. In other studies, analyses rely on a limited set of operational parameters, failing to capture the full complexity of motor behaviour under diverse operating conditions, as discussed in [86].

In this context, the present study distinguishes itself by proposing a more comprehensive solution, both in terms of the diversity of analysed variables and the ability to anticipate failures over a longer horizon. By integrating a broader range of operational data and employing advanced time-series forecasting techniques, the proposed approach enhances predictive maintenance strategies. This contributes to reducing unexpected failures and extending the operational lifespan of three-phase induction motors.

## 3. Methodology

The methodology used in this study began with an exploratory approach to enhance the understanding of factors affecting the operation and performance of three-phase induction motors. We identified the most common failures and their impacts on operational parameters, including stator short circuits, bearing degradation, rotor bar breakage, insulation degradation, load coupling misalignment and poor power quality (harmonics, voltage imbalance or long-duration voltage variations), as reported by [31,32,34]. In the study directed by [2], a graph of the most common problems in a three-phase induction motor can be seen. Given the difficulty of encountering motors in a failure condition in industry and the economic infeasibility of inducing failures in the laboratory, Matlab/Simulink simulation software was employed to collect experimental data [21,22].

### 3.1. Data collection

**Figure 4.**
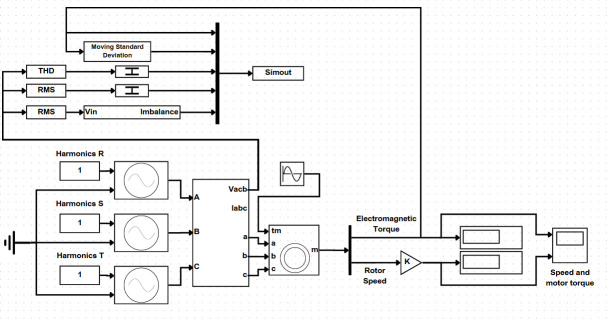
Simulation diagram of a motor analysis system on Simulink.

Three-phase induction motor models were selected for use in the simulation. The powerAsynchronousMachineParams block within Simulink was utilized to obtain their characteristics and nominal data. This block offers a variety of pre-configured motor models, from which motors of 10 HP, 50 HP and 100 HP with a nominal voltage of 460V were chosen. Simulink diagrams were developed to simulate the motor’s behaviour under various conditions, including normal operation and two specific fault conditions. These conditions were selected for further analysis from a pool of six previously identified potential issues. The chosen faults are load coupling and low power quality. The diagram assembly and block configuration were designed for flexibility, allowing the selection of the fault type and timing of its activation through Matlab’s coding interface. This control is made possible by the native integration between Matlab and Simulink. A detailed illustration of the simulation diagram can be found in figure 4.

The selection of data for collection through simulation was based on practical considerations such as ease and cost of data acquisition in a real factory setting. As a result, the data chosen for extraction included steady-state supply voltage, voltage harmonic distortion, voltage imbalance and load torque oscillation (LTO). Simulink simulations were conducted under both nominal and fault conditions with varying input values, generating a significant amount of data that captured motor performance in both healthy and faulty scenarios. Although electromechanical torque is ideal for analysing load changes, the challenges associated with its measurement in real-world settings led to the use of rotational speed as a substitute. However, speed alone is not ideal for fault detection due to the motor’s inertia, so the s.d. of speed, which is more sensitive to oscillations caused by faults, was selected as a key parameter. For each of the measured parameters, limits were set according to ANEEL PRODIST [25] and IEEE and IEC standards [23,24]. These limits are summarized in table 1.

**Table 1.** Measurement parameter limits for three-phase induction motors.

parameters	established limits
voltage harmonics (%)	0 < THDv < 10
voltage imbalance (%)	0 < FD < 3
long-duration voltage variation (%)	0 < *V*_long_ < 10
LTO (%)	0 < LTO < 60

A health index for the induction motor was developed based on established limits. This health index, represented by ‘IS’ in equation (3.1), is calculated by summing the weighted products of various monitored parameters. Each parameter (*Vc*) is determined through a specific logarithmic function and assigned a weight according to ANEEL and IEEE guidelines. The monitored parameters include THDv (odd harmonic distortions of voltage not multiples of 3), *V*_long_ (long-duration voltage variation), FD (voltage imbalance factor) and LTO. These parameters are continuously monitored to generate real-time health index values.


(3.1) $$i_{s}=\sum_{i=1}^{4}{\left(Vc\times \text{weight}\right)}_{i}.$$

The equations for *Vc* related to each parameter, shown in table 2, were determined by considering both the impact of each parameter and the established normative limits. In turn, the selection of weights, which reflects the relative importance of each parameter in the motor’s performance, is explained in further detail in §4.1.

**Table 2.** Weight definition using a logarithmic function.

parameters	*Vc* equation
THDv	log 1.4913 (THDv + 1)
voltage imbalance	log 1.25992 (FD + 1)
long-duration voltage variation	log 1.4913 (*V*_long_+ 1)
LTO	log 1.984057 (LTO + 1)

After calculating the health index ‘IS’, we can extrapolate its results to classify the motor’s health based on the calculated IS. This is useful not only for establishing action plans for different motor health states, as shown in table 3, to address potential issues, but also because supervised learning algorithms require labelled data to learn patterns. With this in mind, the health index was divided into four categories based on the motor’s overall health index, as presented in table 4.

**Table 3.** Recommendations based on the motor health index.

health category	operation recommendations
excellent	motor operating in good conditions.
good	maintain the operation of the equipment. be attentive to possible network deteriorations.
bad	analyse the possibility of quick maintenance in order not to stop the operation of the equipment.
critical	shut down the equipment operation to prevent possible permanent damage.

**Table 4.** Classification of machine health based on the computed health index.

health category	health index
excellent	𝑖_𝑠_ < 3
good	3 ≤ 𝑖_𝑠_ < 6
bad	6 ≤ 𝑖_𝑠_ < 10
critical	𝑖_𝑠_ ≥ 10

### 3.2. Algorithm feeding and training

After data collection, several treatments were applied to prepare it for classification algorithms. These treatments included removing invalid data points, balancing imbalanced datasets and standardizing or normalizing data scales. These steps not only ensured compatibility with the algorithms but also optimized the quality and relevance of the information for the AI models, ultimately improving algorithm accuracy [61]. The system was trained in Python using the following two algorithms: ARIMA and Facebook Prophet via the ‘prophet’ library. For ARIMA/seasonal ARIMA (SARIMA), the ‘pmdarima’ library was utilized, offering a user-friendly interface for fitting both ARIMA and SARIMA models. Its key strength lies in automating various aspects, such as selecting the optimal order for differencing and moving averages within the ARIMA model. Similarly, Facebook Prophet, like pmdarima, is known for its user-friendliness. Its main advantage is that it requires no parameter configuration, making it particularly accessible for beginners.

## 4. Results

### 4.1. Determination of the weights for the health index equation

**Figure 5.**
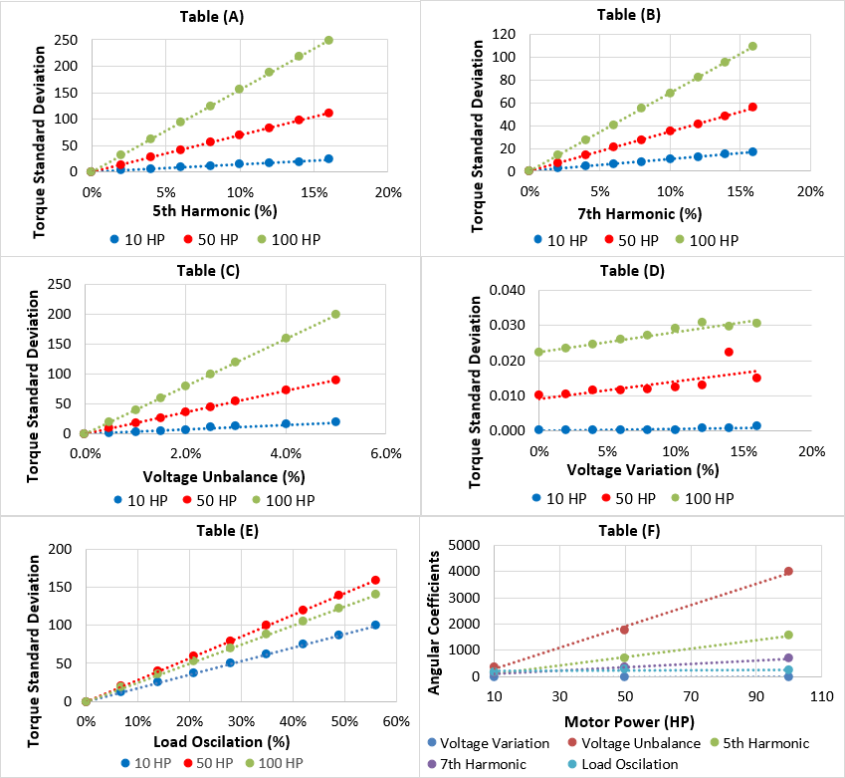
Influence of failures on the torque variation in 10 HP, 50 HP and 100 HP induction motors. (A) Fifth harmonic, (B) seventh harmonic, (C) voltage imbalance, (D) voltage variation, (E) LTO and (F) analysis of the angular coefficient for all failures.

To simulate motor behaviour under various conditions and collect data, three motor powers (10 HP, 50 HP and 100 HP) were chosen. This selection reflects the industry’s wide range of motor applications and ensures applicability to motors of any power due to the spaced-out power values. A Simulink diagram was developed to represent the motor system for simulation (refer to figure 4). This diagram utilized native Simulink blocks to manage switching voltage sources, apply variable loads, capture simulation data and display results in real time. The simulation generated data that could be exported in CSV format, making it suitable for input into the prediction algorithm. The exported data included harmonic voltage distortions, voltage imbalance, long-duration voltage variations and the LTO. Graphs were then generated from this data, as shown in figure 5 on graphs A to E, to illustrate the relationships between motor torque and the analysed disturbances for each simulated motor power. Deviations from the nominal torque curve in these graphs were used as indicators of potential issues in the induction motor.

Analysing the graphs A to E given in figure 5, we can calculate the *R*^²^, also known as the coefficient of determination. *R*^²^ is a crucial statistical tool used in regression models to measure how well the variability of a dependent variable can be explained by the independent variable. This coefficient ranges from 0 to 1, where a value of 0 indicates a weak relationship between the variables in linear regression, suggesting the model has limited ability to account for observed variability. In contrast, an *R*^²^ approaching 1 indicates a strong relationship, implying that the linear regression model fits the data well and explains a significant portion of the variability in the dependent variable [87].

The weight assigned to each disturbance is based on its impact on the motor health index, which is determined by the slope of the line representing the relationship between the disturbance and the index. Steeper slopes indicate a more significant influence on the motor health index. In the case of odd harmonics, the fifth harmonic was selected as it represents the worst-case scenario, but to account for variations across different motor powers, a power-independent weight was calculated. This process involved plotting the angular coefficient (slope) of each disturbance against motor power and deriving a new weight from this graph, ensuring consistency in the weighting regardless of the motor’s power. Graph ‘F’ given in figure 5 illustrates this analysis of the angular coefficient for each disturbance, and the results from both the linear regression of the graphs and the summary of these coefficients can be seen in table 5, facilitating the evaluation of their overall impact on the motor health index.

**Table 5.** Equations of the straight lines for motors with 10 HP, 50 HP and 100 HP.

	10 HP		50 HP		100 HP		generalized for all motor powers	
parameters	line equation	*R*²	line equation	*R*²	line equation	*R*²	line equation	*R*²
**f**ifth harmonic	144.81x + 0.002	1.000	693.8x − 0.0066	1.00	1558.5x − 0.0006	1.00	15.773x − 42.173	0.999
seventh harmonic	103.8x + 0.0022	1.00	346.36x − 0.0042	1.00	682.44x + 0.0096	1.00	6.4413x + 33.997	0.999
voltage imbalance	372.46x − 0.0004	1.00	1778.6x − 0.0029	1.00	3967.2x − 0.0058	1.00	40.099x − 99.169	0.9963
long-duration voltage variation	0.0045x + 0.0002	0.6281	0.048x + 0.0093	0.5144	0.0565x + 0.0225	0.9343	0.0006x + 0.0064	0.823
LTO	177.91x − 0.2545	1.00	249.89x + 0.0157	1.00	283.2x + 0.0926	1.00	0.7397x + 197.55	0.384

As seen from the presented graphs, the analysis of the angular coefficient of the lines revealed that voltage imbalance is the factor with the greatest influence on motor torque. This observation highlights the importance of maintaining balance in the supply voltage to ensure optimized performance of electric motors. Additionally, harmonics, especially the fifth and seventh orders, were identified as secondary contributors. On the other hand, long-duration voltage variation demonstrated a relatively minor influence compared to the other disturbances analysed, with its effect considered negligible for disturbances within the range of voltage values commonly found in the network. The third and ninth harmonics also showed very low influence, primarily because they are zero-sequence harmonics, a characteristic that inherently limits their impact. Therefore, these two harmonics were disregarded.

This finding suggests that to improve operational efficiency and extend motor lifespan, control and maintenance plans should prioritize voltage imbalance and odd harmonics that are not multiples of three. These factors hold more weight in the health index, signifying their greater influence on motor health. Since the function of the weight will always be to amplify the health value, it is necessary to sum the values of the angular coefficients for each disturbance to reflect their respective impacts accurately in the final health index calculation. Thus, obtaining the following weights, as shown in table 6.

**Table 6.** Weight of fault parameters in three-phase induction motors.

parameter	weight
voltage harmonics	1.15
voltage imbalance	1.4
long-duration voltage variation	1
LTO	1.07

### 4.2. Data generation and data feeding

For data generation, specific operational standards were adopted to simulate realistic conditions for the motor’s operation. In this case study, the motor was hypothetically set to operate in a soybean plantation located in Goiás, Brazil. In this context, environmental and operational parameters such as average temperature, precipitation and patterns of soybean planting, harvesting and cultivation were considered. In Goiás, the rainy season typically extends from November to March, during which significant voltage imbalances were factored in. This consideration stems from the tendency for rains to cause faults in the electrical network, potentially leading to the disconnection of one transformer phase. High average temperatures, observed from August to October, were linked to long-duration voltage variation. This association was made because increased loads in the region during hotter months can cause voltage drops. Lastly, the soybean planting cycle was correlated with voltage harmonics, as the activation of a higher number of motors in the network tends to intensify harmonic distortions. For this study, the period from May to July was considered, coinciding with higher motor usage on plantations. The values used to calculate the motor’s health index under these operational conditions are summarized in table 7.

**Table 7.** Table of disturbances included in the simulation.

month	THDv	FD	*V*_long_	LTO
January	0	2.9 < FD < 3.1	0	13.75 < LTO < 27.5
February	0	2.1 < FD < 2.3	0	0
March	0.5 < THDv < 0.6	0	0.7 < *V*_long_ < 0.8	0
April	0.3 < THDv < 0.4	0.4 < FD < 0.5	0	0
May	11 < THDv < 12	0	0	0
June	9 < THDv < 10	0	0	41.25 < LTO < 55
July	7 < THDv < 8	0	0	0
August	0	0	6 < *V*_long_ < 7	0
September	0	0	9 < *V*_long_ < 11	0
October	0	0	7 < *V*_long_ < 8	0
November	0	1.6 < FD < 1.7	0	0
December	0	1.9 < FD < 2.1	0	0

The algorithm used to generate arbitrary data, considering the previously established assumptions, is available in the Dryad Repository [88] under the name ‘Is_Data_Generation.py’. This code aims to build a table with information about the performance of a system over time, considering variables such as voltage, harmonics, unbalance and velocity. These data are generated through simulation and later exported to a file for analysis.

The code is structured into specific functions, each playing a fundamental role in the process:

— *monthly_time_limit()*—Defines the minimum and maximum limits for voltage, harmonics, unbalance and velocity for each month of the year, according to table 7, allowing for the simulation of natural variations in these quantities.
— *generate_random_values()*—Generates random values within the defined ranges, representing simulated system measurements.
— *calculate_health_index()*—Calculates a ‘health index’ for the system based on the simulated values, reflecting its overall condition.
— *generate_data()*—Integrates the previous functions to generate the complete dataset. For each date, it determines the limits for the corresponding month, generates simulated values and calculates the health index.
— *export_to_csv()*—Structures the generated data and exports it to a file compatible with programs like Excel, containing the dates and the calculated health index.

**Figure 6.**
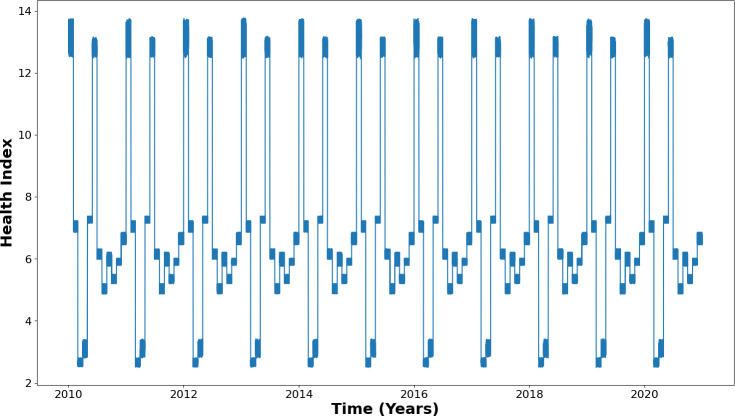
Motor health pattern for 10 years.

Based on these parameters, health indices were calculated, and subsequently, a database was constructed that collected information every hour over a period of 10 years. Figure 6 presents the values assumed by these data over time, with the *Y*-axis corresponding to the motor health index. This approach allows for year-by-year observation of data seasonality, revealing peak health index months (e.g. March) and those with poorer performance (e.g. June). Subsequently, these data were used to train the algorithms, aiming to capture and model these patterns for future value prediction.

### 4.3. ARIMA model

The ARIMA algorithm that processes the data generated by ‘Is_Data_Generation.py’ is available in the Dryad Repository [88] under the name ‘Code_Arima.py’. It analyses the system’s performance over time by examining the *health* variable in the dataset, identifying trends and seasonal patterns and making future predictions using the ARIMA model. The dataset, previously generated through simulation, serves as the foundation for training, testing and validating the predictive model.

The code is structured into the following key steps, each serving a fundamental role in the process:

— *Data loading and visualization*: Reads the generated file, structures the information and creates an initial plot to visualize the complete dataset.
— *Time filtering*: Selects only the data within the 2010−2020 range for focused analysis.
— *Decomposition of the time series*: Breaks the data into three components to better understand its structure:
  — *Trend*: Represents long-term movements in the data.
  — *Seasonality*: Captures recurring patterns at regular intervals.
  — *Random variations*: Accounts for unpredictable fluctuations.
— *Dataset splitting for model training*: Divides the data into the following two subsets:
  — *Training data*: The first 3502 values, representing approximately 96% of the total dataset, used to train the predictive model.
  — *Testing data*: The remaining 4% of the dataset, used to evaluate the model’s accuracy.
— *Model training and forecasting*: Uses an ARIMA model to analyse historical data and generate predictions for the next 90 days.
— *Visualization of results*: Plots the training data, test data and predicted values in a single graph to compare the real and estimated behaviours over time.

**Figure 7.**
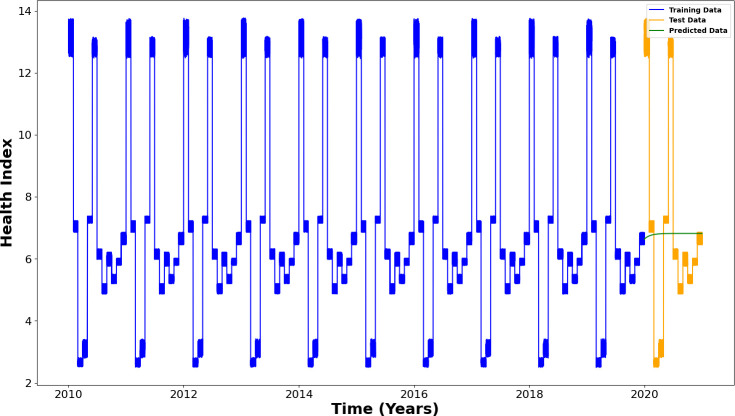
Predicting the future health of a three-phase induction motor using the ARIMA algorithm.

The ARIMA model was trained using 96% of the data, while the remaining 4% was reserved for performance evaluation. The graphical representation of this relationship between training data, test data and health predictions is presented in figure 7.

The ARIMA model, in this case, shows no predictive capability and fails to capture the seasonality within the dataset. Despite being applied, the model could not learn the underlying patterns and failed to adapt to the variations in the health variable over time. This predictive failure can be attributed to the inherent limitations of the model, especially when dealing with complex, nonlinear data, as in the case of this dataset. ARIMA, primarily designed for univariate time series with linear dependencies, struggles with datasets that exhibit stronger seasonal variations, nonlinear trends or other complex patterns that it cannot model effectively.

The model’s failure to capture seasonality may also be due to insufficient seasonal structure in the data or inadequate parameter selection, which may have hindered the recognition of cyclical trends. Additionally, ARIMA models typically require careful parameter tuning to fit the data appropriately, and the automatic selection of these parameters may not have been ideal for this specific dataset.

As a result, the model’s predictions showed no significant improvement over random assumptions, with prediction errors increasing over time. This reinforces the model’s inability to learn the seasonality and dynamics of the dataset.

### 4.4. Facebook Prophet model

The algorithm utilizing Facebook Prophet, similar to the previous one, is also available in the Dryad Repository [88] under the filename ‘Code_Facebook_Prophet.py’. Its structure follows a similar approach, with distinctions specific to the implementation of the Prophet library.

The code is organized in a systematic sequence of steps, which are as follows:

— *Data loading and preprocessing*: The dataset is read from a CSV file and filtered to select the relevant columns (‘Time’ and ‘Health’). The column names are renamed to ‘ds’ (date) and ‘y’ (health), as required by the Prophet model. The data are then sorted chronologically by the ‘ds’ column to ensure proper time order.
— *Model initialization and fitting*: The Prophet model is initialized using the Prophet() function. The model is then trained on the dataset with the fit() method, allowing it to learn the underlying trends and seasonality patterns.
— *Future data generation*: The make_future_dataframe() function extends the dataset by 365 days to create a forecast period. The predict() method is then used to generate predictions for the health variable over this future period.
— *Visualization*: The plot() function visualizes the forecasted data, showing both historical and predicted health values in a time-series plot. This helps in understanding the model’s performance and future trends.
— *Cross-validation and performance metrics*: The cross_validation() function assesses the model’s accuracy by evaluating it over different data subsets, with a specified initial training period, evaluation period and prediction horizon. The performance_metrics() function calculates performance metrics based on the cross-validation results, which help in assessing the model’s accuracy in predicting future values.

**Figure 8.**
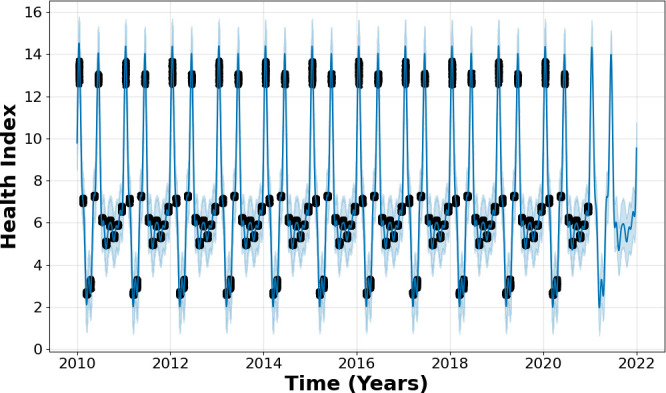
Predicting the future health of a three-phase induction motor using the Facebook Prophet algorithm.

Unlike ARIMA, Facebook Prophet required no configuration adjustments, as it assimilated all the provided data as training input, producing an output that consisted of a temporal sequence of the input data. The algorithm generated multiple possible values for the temporal sequence and graphically represented them, highlighting the most probable values on a chart. This seamless approach, which contrasts with ARIMA’s need for parameter configuration, allowed for a smooth and efficient prediction process. The relationship between the training data, test data and the resulting predictions is visually illustrated in figure 8.

Facebook Prophet demonstrates a significant advantage over ARIMA, showing notable improvement in accurately predicting the health of the induction motor 1 year into the future. The algorithm’s performance is reflected in its low error rates, with a mean squared error of 0.438 and a mean absolute error of 0.467. In the case of Facebook Prophet, its accuracy is evident. Unlike ARIMA, which struggles with increasing errors over longer periods, Prophet maintains a more reliable performance throughout the prediction horizon. Essentially, while ARIMA’s accuracy declines significantly when forecasting beyond short-term periods, Prophet consistently delivers more accurate predictions across the same 1-year timeframe.

## 5. Conclusion

In this study, various factors and potential causes of failures in three-phase induction motors were meticulously examined, analysing the most recurrent failures, their implications and the resulting impacts on the overall performance of the three-phase induction motor. It was observed that voltage imbalance represents the predominant factor, followed by harmonics, s.d. of speed and, lastly, long-duration voltage variation, in that order. Additionally, a method was developed to assess the health of a three-phase induction motor based on power quality data. Concurrently, an innovative method was proposed to predict the motor’s health status, using specialized AI algorithms for time-series forecasting. Among the algorithms used, Facebook Prophet stood out as a notably reliable and accurate choice. Its ease of use, uncomplicated configuration and consistent results exceeded expectations.

However, it is important to note that despite the success in predicting the data, the absence of validation of the method in a real, non-simulated environment represents a significant limitation of this study. The elevated costs associated with testing motors under failure conditions are a major challenge, as operating motors in such states can lead to total failure, making it difficult to conduct these tests in practice. Therefore, future studies should aim to address this gap by establishing partnerships with industries for field testing and leveraging government research and development grants to acquire necessary equipment for laboratory testing.

Furthermore, future research should consider expanding the range of monitored variables to include motor temperature, vibration, noise emissions and startup performance. Incorporating these additional factors into the analysis could provide valuable insights into the motor’s degradation process, improving the reliability of health predictions and advancing the field.

## Data Availability

We have provided all datasets and codes in the electronic supplementary material via the Dryad Repository [88]. Supplementary material is available online [89].

## References

[1] Fuchs EF, Masoumm MAS. 2023 Power quality in power systems and electrical machines, and power electronic drives, 3rd edn. Elsevier. (10.5772/48045)

[2] Leandro E, Lacerda de Oliveira LE de, da Silva JGB, Lambert-Torresm G, da Silv LEB. 2012 Predictive maintenance by electrical signature analysis to induction motors. In Induction motors - modelling and control. InTech. (10.5772/48045)

[3] Duarte B. 2022 Eficiência energética em motores elétricos. GEBRAS. See https://www.gebras.com/gebras/news-item.php?id=1120 (accessed 27 May 2024).

[4] IEA - International Energy Agency. 2023 Quarterly average wholesale prices for selected regions 2019-2025. IEA. See https://www.iea.org/reports/electricity-2024.

[5] Polenghi A, Roda I, Macchi M, Pozzetti A. 2023 A methodology to boost data-driven decision-making process for a modern maintenance practice. Prod. Plan. Control **34**, 1333–1349. (10.1080/09537287.2021.2010823)

[6] Sánchez RV, Macancela JC, Ortega LR, Cabrera D, García Márquez FP, Cerrada M. 2024 Evaluation of hand-crafted feature extraction for fault diagnosis in rotating machinery: a survey. Sensors **24**, 5400. (10.3390/s24165400) 39205095 PMC11360600

[7] Tantardini M, Portioli-Staudacher A, Macchi M. 2014 A model for considering the impact of rescheduling planned maintenance activities in a maintenance service contract. Prod. Plan. Control **25**, 241–259. (10.1080/09537287.2012.665094)

[8] Dowlatshahi S. 2009 The maquiladora industry and equipment maintenance: an industry-based perspective. Prod. Plan. Control **20**, 227–241. (10.1080/09537280902843573)

[9] Fornasiero R, Zangiacomi A, Sorlini M. 2012 A cost evaluation approach for trucks maintenance planning. Prod. Plan. Control **23**, 171–182. (10.1080/09537287.2011.591641)

[10] Yan J, Koç M, Lee J. 2004 A prognostic algorithm for machine performance assessment and its application. In Production planning and control pp. 796–801 (10.1080/09537280412331309208)

[11] Huang R, Xi L, Lee J, Liu CR. 2005 The framework, impact and commercial prospects of a new predictive maintenance system: intelligent maintenance system. Prod. Plan. Control **16**, 652–664. (10.1080/09537280500205837)

[12] Picciolo G, Galli F, Biamonti A, Magni P. 2008 Determining the maximum periodic inspection interval for medium voltage motors using a Markov model. Prod. Plan. Control **19**, 356–364. (10.1080/09537280802034224)

[13] Kavusturucu A, Gupta S. 1998 Tandem manufacturing systems with machine vacations. Prod. Plan. Control **9**, 494–503. (10.1080/095372898233975)

[14] Gopalakrishnan M, Subramaniyan M, Skoogh A. 2022 Data-driven machine criticality assessment–maintenance decision support for increased productivity. Prod. Plan. Control **33**, 1–19. (10.1080/09537287.2020.1817601)

[15] Farrar CR, Lieven NAJ. 2007 Damage prognosis: the future of structural health monitoring. Phil. Trans. R. Soc. A **365**, 623–632. (10.1098/rsta.2006.1927) 17255054

[16] Abualsauod EH. 2023 Machine learning based fault detection approach to enhance quality control in smart manufacturing. Production Planning & Control **Special Issue**, 1–9. (10.1080/09537287.2023.2175736)

[17] Helo P, Hao Y. 2022 Artificial intelligence in operations management and supply chain management: an exploratory case study. Prod. Plan. Control **33**, 1573–1590. (10.1080/09537287.2021.1882690)

[18] Li C, Hu JW. 2012 A new ARIMA-based neuro-fuzzy approach and swarm intelligence for time series forecasting. Eng. Appl. Artif. Intell. **25**, 295–308. (10.1016/j.engappai.2011.10.005)

[19] Ghimire S, Deo RC, Ali Pourmousavi S, Casillas-Pérez D, Salcedo-Sanz S. 2024 Point-based and probabilistic electricity demand prediction with a neural facebook prophet and kernel density estimation model. Eng. Appl. Artif. Intell. **135**, 108702. (10.1016/j.engappai.2024.108702)

[20] Vu KM. 2007 The arima and varima time series: their modelings, analyses and applications, 1st edn. Ottawa, Canada: AuLac Technologies.

[21] Le-Huy H 2001 Modeling and simulation of electrical drives using MATLAB/Simulink and Power System Blockset. In IECON’01. 27th Annual Conference of the IEEE Industrial Electronics Society, Denver, CO, USA, pp. 1603–1611. IEEE. (10.1109/IECON.2001.975530)

[22] Sharma SK, Manna MS. 2022 Performance analysis of universal motor based on matlab simulation. In 2022 International Conference for Advancement in Technology (ICONAT), Goa, India, pp. 1–4. IEEE. (10.1109/ICONAT53423.2022.9726017)

[23] IEC. 2022 IEC 600341:2022: rotating electrical machines - Part 1: rating and performance. Geneva, Switzerland: IEC. See https://webstore.iec.ch/en/publication/65446.

[24] IEC. 1998 IEC 61000-3-4:1998 electromagnetic compatibility (EMC) - Part 3-4: limits - limitation of emission of harmonic currents in low-voltage power supply systems for equipment with rated current greater than 16 A. IEC. See https://webstore.iec.ch/en/publication/4151.

[25] Prodist. 2020 Procedimentos de distribuição de energia elétrica no sistema elétrico nacional – PRODIST - Módulo 8 - Qualidade da Energia Elétrica. Brazil: ANEEL - Brazilian Electricity Agency. See https://www2.aneel.gov.br/cedoc/aren2020888_prodist_modulo_8_v11.pdf.

[26] WEG SA. 2023 Specification guide electric motors. WEG. See https://static.weg.net/medias/downloadcenter/ha0/h5f/WEG-motors-specification-of-electric-motors-50039409-brochure-english-web.pdf.

[27] Fitzgerald A, Kingsley C. 2014 Electric machinery, 7th edn. New York, NY, USA: McGraw-Hill.

[28] Zhong J, Mao H, Tang W, Qin A, Sun K. 2023 Intelligent fault diagnosis scheme for rotating machinery based on momentum contrastive bi-tuning framework. Eng. Appl. Artif. Intell. **122**, 106100. (10.1016/j.engappai.2023.106100)

[29] Engelbrecht AP, Grobler J, Langeveld J. 2019 Set based particle swarm optimization for the feature selection problem. Eng. Appl. Artif. Intell. **85**, 324–336. (10.1016/j.engappai.2019.06.008)

[30] Irgat E, Unsal A, Canseven HT. 2021 Detection of Eccentricity Faults of Induction Motors Based on Decision Trees. In 2021 13th International Conference on Electrical and Electronics Engineering (ELECO), Bursa, Turkey, pp. 435–439. IEEE. (10.23919/ELECO54474.2021.9677809)

[31] Tran VT, Yang BS, Oh MS, Tan ACC. 2009 Fault diagnosis of induction motor based on decision trees and adaptive neuro-fuzzy inference. Expert Syst. Appl. **36**, 1840–1849. (10.1016/j.eswa.2007.12.010)

[32] Zhang J, Hu D, Yang T, Zhou H, Li X. 2024 A time series and deep fusion framework for rotating machinery fault diagnosis. Eng. Appl. Artif. Intell. **128**, 107456. (10.1016/j.engappai.2023.107456)

[33] AlShorman O, Irfan M, Abdelrahman RB, Masadeh M, Alshorman A, Sheikh MA, Saad N, Rahman S. 2024 Advancements in condition monitoring and fault diagnosis of rotating machinery: a comprehensive review of image-based intelligent techniques for induction motors. Eng. Appl. Artif. Intell. **130**, 107724. (10.1016/j.engappai.2023.107724)

[34] Gawde S, Patil S, Kumar S, Kamat P, Kotecha K, Abraham A. 2023 Multi-fault diagnosis of industrial rotating machines using data-driven approach: a review of two decades of research. Eng. Appl. Artif. Intell. **123**, 106139. (10.1016/j.engappai.2023.106139)

[35] Hassan OE, Amer M, Abdelsalam AK, Williams BW. 2018 Induction motor broken rotor bar fault detection techniques based on fault signature analysis – a review. IET Electr. Power Appl. **12**, 895–907. (10.1049/iet-epa.2018.0054)

[36] Santoso S. 2012 Fundamentals of electric power quality. Washington, United States: CreateSpace Independent Publishing Platform.

[37] Singh B, Chandra A, Al-Haddad K. 2014 Power quality: problems and mitigation techniques. New Jersey, United States: Wiley. (10.1002/9781118922064)

[38] Dugan R, McGranaghan M, Santoso S, Beaty H. 2002 Electrical power systems quality, 3rd edn. New York, United States: McGraw-Hill Professional.

[39] Azevedo G de, Romão EC, Menegatti CR. 2019 Correção de distorções harmônicas em sistemas elétricos através de interferência destrutiva, Rev. Bras. De Ensino De Física. (10.1590/1806-9126-rbef-2018-0278). See https://www.scielo.br/j/rbef/a/TBxQnsqq3kgctLCPzMkZ3rw/.

[40] Padmanaban S, Sharmeela C, Holm-Nielsen J, Sivaraman P. 2020 Power quality in modern power systems. Oxford, UK: Academic Press.

[41] Asad B, Vaimann T, Kallaste A, Belahcen A. 2018 Harmonic Spectrum Analysis of Induction Motor With Broken Rotor Bar Fault. In 2018 IEEE 59th International Scientific Conference on Power and Electrical Engineering of Riga Technical University (RTUCON), Riga, Latvia. Institute of Electrical and Electronics Engineers Inc. (10.1109/RTUCON.2018.8659842)

[42] Fuchs E, Masoum M. 2015 Power quality in power systems and electrical machines, 2nd edn. Oxford, UK: Academic Press.

[43] González de la Rosa JJ, Donsión M. 2020 Analysis for power quality monitoring. Basel, Switzerland: MDPI AG. (10.3390/en13030514). See https://www.mdpi.com/books/reprint/2294-analysis-for-power-quality-monitoring.

[44] Mamede D. 2020 Proteção de sistemas elétricos de potência. Rio de Janeiro, RJ: LTC.

[45] Sekhar AS, Prabhu BS. 1995 Effects of coupling misalignment on vibrations of rotating machinery. J. Sound Vib. **185**, 655–671. (10.1006/jsvi.1995.0407)

[46] Ackson R, Aguire C. 1968 Noise of electrical machines. Phil. Trans. R. Soc. Lond. A Math. Phy Sci. **263**, 413–423.

[47] Li J, Ying Y, Ren Y, Xu S, Bi D, Chen X, Xu Y. 2019 Research on rolling bearing fault diagnosis based on multi-dimensional feature extraction and evidence fusion theory. R. Soc. Open Sci. **6**, 181488. (10.1098/rsos.181488) 30891276 PMC6408408

[48] Mikkili S, Panda A. 2018 Power quality issues: current harmonics. Florida, United States: CRC Press.

[49] Yang C, Wang H, Gao Z, Cui X. 2018 Improving rolling bearing online fault diagnostic performance based on multi-dimensional characteristics. R. Soc. Open Sci. **5**, 180066. (10.1098/rsos.180066) 29892444 PMC5990754

[50] Sadhu A, Prakash G, Narasimhan S. 2017 A hybrid hidden Markov model towards fault detection of rotating components. J. Vib. Control **23**, 3175–3195. (10.1177/1077546315627934)

[51] Singh SR, Dhami SS, Pabla BS. 2018 Development of low-cost non-contact structural health monitoring system for rotating machinery. R. Soc. Open Sci. **5**, 172430. (10.1098/rsos.172430) 30110455 PMC6030280

[52] Kelch CK, Grover PE. 1996 Using thermography to detect misalignment in coupled equipment. In Thermosense XVIII: An International Conference on Thermal Sensing and Imaging Diagnostic Applications, pp. 91–100. Washington, United States: SPIE. (10.1117/12.235371)

[53] Lopez-Perez D, Antonino-Daviu J. 2017 Application of infrared thermography to failure detection in industrial induction motors: case stories. IEEE Trans. Ind. Appl. **53**, 1901–1908. (10.1109/tia.2017.2655008)

[54] Bennett B. 2015 Unbalanced voltage supply - the damaging effects on three phase induction motors and rectifiers. Asea Brown Boveri. See https://search.abb.com/library/Download.aspx?DocumentID=2UCD401218-P&LanguageCode=en&DocumentPartId=&Action=Launch.

[55] Lima E. 2016 Diagnóstico de motores de indução trifásicos operando em redes desequilibradas e distorcidas. Itajubá-MG, Brazil: Universidade Federal de Itajubá. See https://repositorio.unifei.edu.br/jspui/handle/123456789/642.

[56] Chauhan S, Singh SB. 2019 Effects of voltage unbalance and harmonics on 3-phase induction motor during the condition of undervoltage and overvoltage. In 2019 6th International Conference on Signal Processing and Integrated Networks (SPIN), Noida, India, pp. 1141–1146. IEEE. (10.1109/SPIN.2019.8711753)

[57] Dhami DSS, Pabla BS. 2017 Non-contact incipient fault diagnosis method of fixed-axis gearbox based on CEEMDAN. R. Soc. Open Sci. **4**, 170616. (10.1098/rsos.170616) 28879003 PMC5579119

[58] Bonnett AH. The impact that voltage and frequency variations have on AC induction motor performance and life in accordance with NEMA MG-1 standards. In Conference Record of 1999 Annual Pulp and Paper Industry Technical Conference, Seattle, WA, USA, pp. 16–26. IEEE. (10.1109/PAPCON.1999.779341)

[59] Chandra Sekhar Reddy M, Sekhar AS. 2015 Detection and monitoring of coupling misalignment in rotors using torque measurements. Measurement **61**, 111–122. (10.1016/j.measurement.2014.10.031)

[60] Gerada C, Bradley KJ, Sumner M, Wheeler P, Pickering S, Clare J, Whitley C, Towers G. The implications of winding faults in induction motor drives. In Conference Record of the 2004 IEEE Industry Applications Conference, 2004. 39th IAS Annual Meeting, Seattle, WA, USA, pp. 2506–2513. IEEE. (10.1109/IAS.2004.1348827)

[61] Russel S, Norvig P. 2013 Artificial intelligence - a modern approach. Hoboken, NJ, USA: Pearson Education.

[62] Warke V, Kumar S, Bongale A, Kamat P, Kotecha K, Selvachandran G, Abraham A. 2024 Improving the useful life of tools using active vibration control through data-driven approaches: a systematic literature review. Eng. Appl. Artif. Intell. **128**, 107367. (10.1016/j.engappai.2023.107367)

[63] Sánchez RV, Lucero P, Vásquez RE, Cerrada M, Macancela JC, Cabrera D. 2018 Feature ranking for multi-fault diagnosis of rotating machinery by using random forest and KNN. J. Intell. Fuzzy Syst. **34**, 3463–3473. (10.3233/jifs-169526)

[64] Pacheco F, Drimus A, Duggen L, Cerrada M, Cabrera D, Sanchez RV. 2022 Deep ensemble-based classifier for transfer learning in rotating machinery fault diagnosis. IEEE Access **10**, 29778–29787. (10.1109/access.2022.3158023)

[65] Sanchez RV, Lucero P, Macancela JC, Cerrada M, Vasquez RE, Pacheco F. 2017 Multi-fault Diagnosis of Rotating Machinery by Using Feature Ranking Methods and SVM-based Classifiers. In 2017 International Conference on Sensing, Diagnostics, Prognostics and Control (SDPC), Shanghai, pp. 105–110. IEEE. (10.1109/SDPC.2017.29)

[66] Pacheco F, Cerrada M, Sanchez RV, Cabrera D, Li C, de Oliveira JV. 2016 A methodological framework using statistical tests for comparing machine learning based models applied to fault diagnosis in rotating machinery. In 2016 IEEE Latin American Conference on Computational Intelligence (LA-CCI), Cartagena, Colombia, pp. 1–6. IEEE. (10.1109/LA-CCI.2016.7885715)

[67] Li C, Sánchez RV, Zurita G, Cerrada M, Cabrera D. 2016 Fault diagnosis for rotating machinery using vibration measurement deep statistical feature learning. Sensors **16**, 895. (10.3390/s16060895) 27322273 PMC4934321

[68] Gao F, Shao X. 2024 Electricity consumption prediction based on a dynamic decomposition-denoising-ensemble approach. Eng. Appl. Artif. Intell. **133**, 108521. (10.1016/j.engappai.2024.108521)

[69] Costa RL de C. 2022 Convolutional-LSTM networks and generalization in forecasting of household photovoltaic generation. Eng. Appl. Artif. Intell. **116**, 105458. (10.1016/j.engappai.2022.105458)

[70] Arena S, Florian E, Sgarbossa F, Sølvsberg E, Zennaro I. 2024 A conceptual framework for machine learning algorithm selection for predictive maintenance. Eng. Appl. Artif. Intell. **133**, 108340. (10.1016/j.engappai.2024.108340)

[71] Manjare AA, Patil BG. 2021 A review: condition based techniques and predictive maintenance for motor. In 2021 International Conference on Artificial Intelligence and Smart Systems (ICAIS), Coimbatore, India, pp. 807–813. Institute of Electrical and Electronics Engineers Inc. (10.1109/ICAIS50930.2021.9395903). https://ieeexplore.ieee.org/xpl/mostRecentIssue.jsp?punumber=9395728.

[72] Mykoniatis K. 2020 A real-time condition monitoring and maintenance management system for low voltage industrial motors using internet-of-things. Procedia Manuf. **42**, 450–456. (10.1016/j.promfg.2020.02.050)

[73] Nikfar M, Bitencourt J, Mykoniatis K. 2022 A two-phase machine learning approach for predictive maintenance of low voltage industrial motors. Procedia Comput. Sci. **200**, 111–120. (10.1016/j.procs.2022.01.210)

[74] Mohammed N, Abdulateef O, Hamad A. 2023 An IoT and machine learning-based predictive maintenance system for electrical motors. J. Eur. Des Syst. Autom. **56**, 651–656. (10.18280/jesa.560414)

[75] Patel RA, Bhalja BR. 2016 Condition monitoring and fault diagnosis of induction motor using support vector machine. Electr. Power Components Syst. **44**, 683–692. (10.1080/15325008.2015.1131762)

[76] Satria H, Dhabliya D, Srisainath R, Edwin Prabhakar PB, Sivakamasundari S. 2023 Designing a predictive maintenance-based algorithm for DC motor fault detection and classification. In 2023 3rd International Conference on Technological Advancements in Computational Sciences (ICTACS), Tashkent, Uzbekistan, pp. 212–218. Institute of Electrical and Electronics Engineers Inc. (10.1109/ICTACS59847.2023.10390249)

[77] Herzog T, Bartecki K. 2024 Predictive maintenance for electrical motors: current approach and usage of artificial intelligence algorithms. In 2024 28th International Conference on Methods and Models in Automation and Robotics (MMAR), Poland, pp. 494–498. Institute of Electrical and Electronics Engineers Inc. (10.1109/MMAR62187.2024.10680808)

[78] Bundasak S, Wittayasirikul P. 2022 Predictive maintenance using AI for motor health prediction system. In 2022 International Electrical Engineering Congress (iEECON), Khon Kaen, Thailand. Institute of Electrical and Electronics Engineers Inc. (10.1109/iEECON53204.2022.9741620)

[79] Liu Z, Yuan T, Zhou X, Yuan X, Wang Y, Zhang X. 2020 Research on Predictive Maintenance Technology of Stepping Motor Based on Load Value Analysis. In 2020 Chinese Automation Congress (CAC), Shanghai, China, p. 7122. Institute of Electrical and Electronics Engineers. (10.1109/CAC51589.2020.9327408). https://ieeexplore.ieee.org/xpl/mostRecentIssue.jsp?punumber=9326315.

[80] Cakir M, Guvenc MA, Mistikoglu S. 2021 The experimental application of popular machine learning algorithms on predictive maintenance and the design of IIoT based condition monitoring system. Comput. Ind. Eng. **151**, 106948. (10.1016/j.cie.2020.106948)

[81] Antonino-Daviu J, Dunai L, Osornio-Rios RA, Zamudio-Ramirez I. 2023 Use of smart diagnosis systems for education on electric motors predictive maintenance. In 2023 IEEE 10th International Conference on E-Learning in Industrial Electronics (ICELIE), Singapore. Institute of Electrical and Electronics Engineers. (10.1109/ICELIE58531.2023.10313098)

[82] Yousuf M, Alsuwian T, Amin AA, Fareed S, Hamza M. 2024 IoT-based health monitoring and fault detection of industrial AC induction motor for efficient predictive maintenance. Meas. Control **57**, 1146–1160. (10.1177/00202940241231473)

[83] Implementing condition based maintenance using modelling and simulation. In press.

[84] Bhandari M, Silwal B. 2022 Development of Machine Learning Model Applied to Industrial Motors for Predictive Maintenance. In 2022 International Interdisciplinary Humanitarian Conference for Sustainability (IIHC), Bengaluru, India, pp. 1632–1635. Institute of Electrical and Electronics Engineers Inc. (10.1109/IIHC55949.2022.10060358)

[85] Jayaswal B, Agrawal SS, Jain S, Singh R, Kashyap K, Chauhan P. 2023 Predictive maintenance system for rotating machinery onboard ships for detecting performance degradation. Scalable Comput. **24**, 1231–1240. (10.12694/scpe.v24i4.2016)

[86] Hanifi S, Alkali B, Lindsay G, Waters M, McGlinchey D. 2024 Measurements: Sensors. Advancements in predictive maintenance modelling for industrial electrical motors: Integrating machine learning and sensor technologies. (10.1016/j.measen.2024.101473)

[87] DeMARIS A. 2002 Explained variance in logistic regression. Sociol. Methods Res. **31**, 27–74. (10.1177/0049124102031001002)

[88] Aires F, Galeno G. 2024 Dataset - enhancing three-phase induction motor reliability with health index and AI-driven predictive maintenance. Dryad Digital Repository. (10.5061/dryad.x0k6djhvd) PMC12115838 40438546

[89] Aires FL, Galeno GD, Belchior FN, Oliveira AM, Hunt JD. 2025. Supplementary Material from: Enhancing Three-Phase Induction Motor Reliability with Health Index and Ai-Driven Predictive Maintenance. FigShare (10.6084/m9.figshare.c.7829234) PMC12115838 40438546

